# Specificity of anxiety-phobic states in the structure of psychosomatic relations of hypertension as a criterion for differentiation of psychotherapeutic approaches

**DOI:** 10.1192/j.eurpsy.2025.1243

**Published:** 2025-08-26

**Authors:** M. Markova, I. Sarvir, A. Avramenko, S. Martynenko

**Affiliations:** 1 Sexology, Psychotherapy and Medical Psychology, Kharkiv National Medical University, Kharkiv, Ukraine

## Abstract

**Introduction:**

The study of anxiety-phobic states as an integral part of psychosomatic relationships in cardiovascular pathology allows us to detail the pathogenetic mechanisms of formation, develop approaches to early diagnostics, psychotherapy and psychoprophylaxis, which is of unconditional medical and social importance in solving this problem.

**Objectives:**

Improving the effectiveness of therapy for patients with hypertension, including in combination with ischemic heart disease (IHD), with anxiety states based on the personalization of psychotherapy.

**Methods:**

The survey included the use of clinical, socio-demographic, instrumental, clinical-psychopathological, psychodiagnostic and statistical methods.

**Results:**

The study sample consisted of 120 patients who were treated at the Kharkiv Clinical Hospital on Railway Transport No. 1, JSC Ukrzaliznytsia. It was revealed that the main psychopathological variants of anxiety states in patients with hypertension are anxiety and combined fears (biological, situational and social, enhanced by psychotraumatic events related to war). The intensity and spectrum of anxiety states have a direct correlation with the duration, severity of the somatic disease and the severity of psychopathological symptoms.

For patients with hypertension with IHD, the dominant fear is typically focused on their own biological well-being (90.5%), for patients with hypertension – on the biological and social well-being of significant others (74,4%).

The dynamics of anxiety states are different: in 57,7% of patients with hypertension, fear initially develops as a result of traumatic social or situational factors, in 69,1% of patients with hypertension with IHD, unreasonable anxiety acquired a plot due to somatic distress. Psychopathological symptoms in patients with hypertension with anxiolytic states are represented by pronounced asthenic manifestations (95,8%) in combination with hypochondriacal (37,5%), depressive (24,2%), and vegetative (15,0%).

A differentiated psychotherapeutic model for the correction of psychosomatic relationships has been developed, aimed at deactualization of fears, reduction of anxiety, elimination of psychopathological manifestations, improvement of the somatic state, improvement of the quality of life and social adaptation.

**Conclusions:**

The application of the developed model has shown its high efficiency in the complex therapy of patients with hypertension.

**Disclosure of Interest:**

None Declared

